# Molecular recognition of wood polyphenols by phase II detoxification enzymes of the white rot *Trametes versicolor*

**DOI:** 10.1038/s41598-018-26601-3

**Published:** 2018-05-31

**Authors:** Mathieu Schwartz, Thomas Perrot, Emmanuel Aubert, Stéphane Dumarçay, Frédérique Favier, Philippe Gérardin, Mélanie Morel-Rouhier, Guillermo Mulliert, Fanny Saiag, Claude Didierjean, Eric Gelhaye

**Affiliations:** 1 Université de Lorraine, CNRS, CRM2 Nancy, France; 2 Université de Lorraine, INRA, IAM, Nancy, France; 3 Université de Lorraine, LERMAB, Nancy, France

## Abstract

Wood decay fungi have complex detoxification systems that enable them to cope with secondary metabolites produced by plants. Although the number of genes encoding for glutathione transferases is especially expanded in lignolytic fungi, little is known about their target molecules. In this study, by combining biochemical, enzymatic and structural approaches, interactions between polyphenols and six glutathione transferases from the white-rot fungus *Trametes versicolor* have been demonstrated. Two isoforms, named TvGSTO3S and TvGSTO6S have been deeply studied at the structural level. Each isoform shows two distinct ligand-binding sites, a narrow L-site at the dimer interface and a peculiar deep hydrophobic H-site. In TvGSTO3S, the latter appears optimized for aromatic ligand binding such as hydroxybenzophenones. Affinity crystallography revealed that this H-site retains the flavonoid dihydrowogonin from a partially purified wild-cherry extract. Besides, TvGSTO6S binds two molecules of the flavonoid naringenin in the L-site. These data suggest that TvGSTO isoforms could interact with plant polyphenols released during wood degradation.

## Introduction

The microbial degradation of wood has been extensively studied due to its importance in organic matter recycling and its potential valorisation in many industrial domains. This degradation is mainly mediated by fungi and in particular by white-rot fungi which are able to degrade and mineralize all the wood components. Indeed, as early as in the middle of last century, this functional trait has been correlated to the ability of these fungi to secrete extracellular enzymatic systems able to degrade wood polymers^1^. Thanks to the recent release of more than fifty fungal genomes, comparative genomic approaches have confirmed this correlation^2,3^. Beyond these extracellular systems, recent studies have also confirmed the importance of intracellular detoxification systems in the fungal wood degradation process^4^. These systems were thought to play essential roles in wood degradation. They allow fungi to (i) catabolize the oxidized compounds that result from lignin oxidation^5^, and (ii) cope with wood anti-microbial compounds, such as flavonoids, stilbenes or terpenes^6,7^. The efficiency of these intracellular detoxification systems seems to be linked to the expansion of multigenic families involved in the oxidation phase such as cytochrome P450 mono-oxygenases and in the conjugation phase such as glutathione transferases (GSTs)^6,8^. Similarly, such expansions are also found in herbivorous insects, where these multigenic families play key functions in the detoxification of plant defense chemicals and also in the evolution of metabolic resistance to chemical insecticides^9–11^.

Until now, in wood-decaying fungi, comparative genomic, biochemical, structural or physiological approaches gave only few insights into the function and specificity of these enzymes in the wood degradation process. This lack of knowledge is mainly due to the absence of specific substrates that would allow discrimination between the isoforms. The expansion of the GST family in these fungi mainly concerns three phylogenetic classes, named GSTFuA, Ure2p and GST Omega^12^. We had suggested that the fungal-specific GSTFuA class could be involved in the catabolism of lignin derived molecules^13^. A recent study confirmed that an isoform from *Dichomitus squalens* (*Ds*-GST1) selectively cleaves the β-O-4 aryl ether bond of a dimeric lignin model leading to a glutathione derivative^14^. Concerning the Ure2p class, it can be divided in two subclasses with distinct structural and biochemical properties. Ure2pAs possess the classic GSH transferase activity while Ure2pBs display a deglutathionylation activity (Ure2pB)^15^. Interestingly, bacterial orthologs of Ure2pB (named GST Nu) act as glutathione lyases in breaking the β-aryl ether bond of lignin^16^. GSTOs are involved in detoxification pathways via deglutathionylation reactions^17^ and two GSTO isoforms of *Phanerochaete chrysosporium* (PcGSTO3 and PcGSTO4) bind terpenes^18^. In *Trametes versicolor*, some GSTO isoforms interact with different wood extractives^19^. These interactions could give insights into the chemical composition of the extracts.

To further explore this issue, we have conducted biochemical and crystallographic studies on the interactions of six GSTOs from *T. versicolor* with chemical libraries. We showed that these GSTOs exhibit distinct affinity patterns, particularly with benzophenones and flavonoids. An affinity crystallography approach allowed the isolation of a flavonoid from a partially-purified wild-cherry tree extract. This ligand specific to one GSTO isoform was characterized as dihydrowogonin using multiple approaches. All these results and the recent literature support the conclusion that GSTs of this class interact with wood polyphenolic compounds.

## Results and Discussion

### TvGSTO3S interacts with hydroxybenzophenones

Thermal shift assay (TSA) is a high-throughput ligand-screening method based on the modification of protein thermal denaturation. According to a gradient of temperature, the denaturation is followed by monitoring fluorescence enhancement of a probe (SYPRO Orange) that binds to protein hydrophobic patches upon denaturation process. This TSA method has been successfully used to detect interactions between proteins and libraries of molecules^20^. It allowed us to identify chemical families of compounds that interact with TvGSTOs and prompted us to investigate more deeply the case of TvGSTO3S with hydroxybenzophenones (HBPs), in particular by conducting a structural analysis of protein-ligand complexes.

First, the interactions between a chemical library of 27 compounds and six TvGSTOs were explored using TSA (Supplementary Fig. S1). The tested compounds were chosen either for their presence in wood or their reactivity with GSTs^19^. The six TvGSTOs (named TvGSTO1S to TvGSTO6S) used in this study are representatives of the twelve TvGSTOs that have a catalytic serine, while four others have a cysteine instead. The obtained results show patterns of interaction that distinguish each isoform from the others. Indeed, a few compounds significantly increased the stability of TvGSTO1S, 3S and 6S, *i.e*. they show a variation of the denaturation temperature ∆Td > 5 °C. On the contrary, most chemical compounds induced a destabilizing effect on TvGSTO5S and 2S (∆Td < 0 °C), whereas the chemical library had little impact on TvGSTO4S thermal stability. In the case of TvGSTO3S, the compounds that increased protein stability all belong to the same chemical family, namely hydroxybenzophenones (HBPs). Benzophenones are present in plant extracts^21^ and also in wood extractives, for example in oak heartwood^22^.

A set of commercially available HBPs with various numbers and positions of hydroxylation on their rings A and B (Table 1) was tested by using TSA and the six TvGSTOs. It confirmed that TvGSTO3S has a significant affinity for HBPs. For this isoform, a group of molecules (2,4-, 3,4-, 2,3,4- and 2,4,4′- HBPs) corresponding to compounds with at least two hydroxyl groups on ring A and no more than one hydroxylation on ring B stand out for their causing large increases in the melting temperature of TvGSTO3S. Oppositely, 2,2′-, 4,4′-, 2,2′,4,4′- HBPs and the unsubstituted benzophenone had little or no effect on the thermal stability of TvGSTO3S. The replacement of the 4-hydroxyl group of 2,4-HBP by a methoxy group nullified the observed thermal shift.

**Table 1:** Summary of the results obtained for TvGSTOs with benzophenone compounds by thermal-shift assays.

Molecules	Structures	TvGSTO1S	TvGSTO2S	TvGSTO3S	TvGSTO4S	TvGSTO5S	TvGSTO6S
Benzophenone		n.s.	n.s.	n.s.	n.s.	n.s.	n.s.
2,2′-Dihydroxy benzophenone		n.s.	n.s.	n.s.	n.s.	n.s.	n.s.
2,4-Dihydroxy benzophenone		2.05 °C	2.56 °C	5.69 °C	n.s.	n.s.	1.63 °C
3,4-Dihydroxy benzophenone		0.98 °C	n.s.	4.36 °C	2.77 °C	n.s.	n.s.
4,4′-Dihydroxy benzophenone		n.s.	n.s.	1.67 °C	n.s.	n.s.	n.s.
2,3,4-Trihydroxy benzophenone		1.13 °C	2.92 °C	4.96 °C	1.76 °C	−12.89 °C	4.15 °C
2,4,4′-Trihydroxy benzophenone		2.74 °C	2.03 °C	2.87 °C	n.s.	n.s.	1.28 °C
2,2′,4,4′-Tetrahydroxy benzophenone		3.74 °C	0.74 °C	n.s.	n.s.	n.s.	3.40 °C
2-Hydroxy-4-methoxy benzophenone		n.s.	n.s.	n.s.	n.s.	n.s.	n.s.

A ∆Td value is only given if the denaturation temperature is significantly modified in the presence of compounds, with respect to incubation with DMSO only. “n.s.” means that the denaturation temperature has not changed significantly.

**Figure 1:**
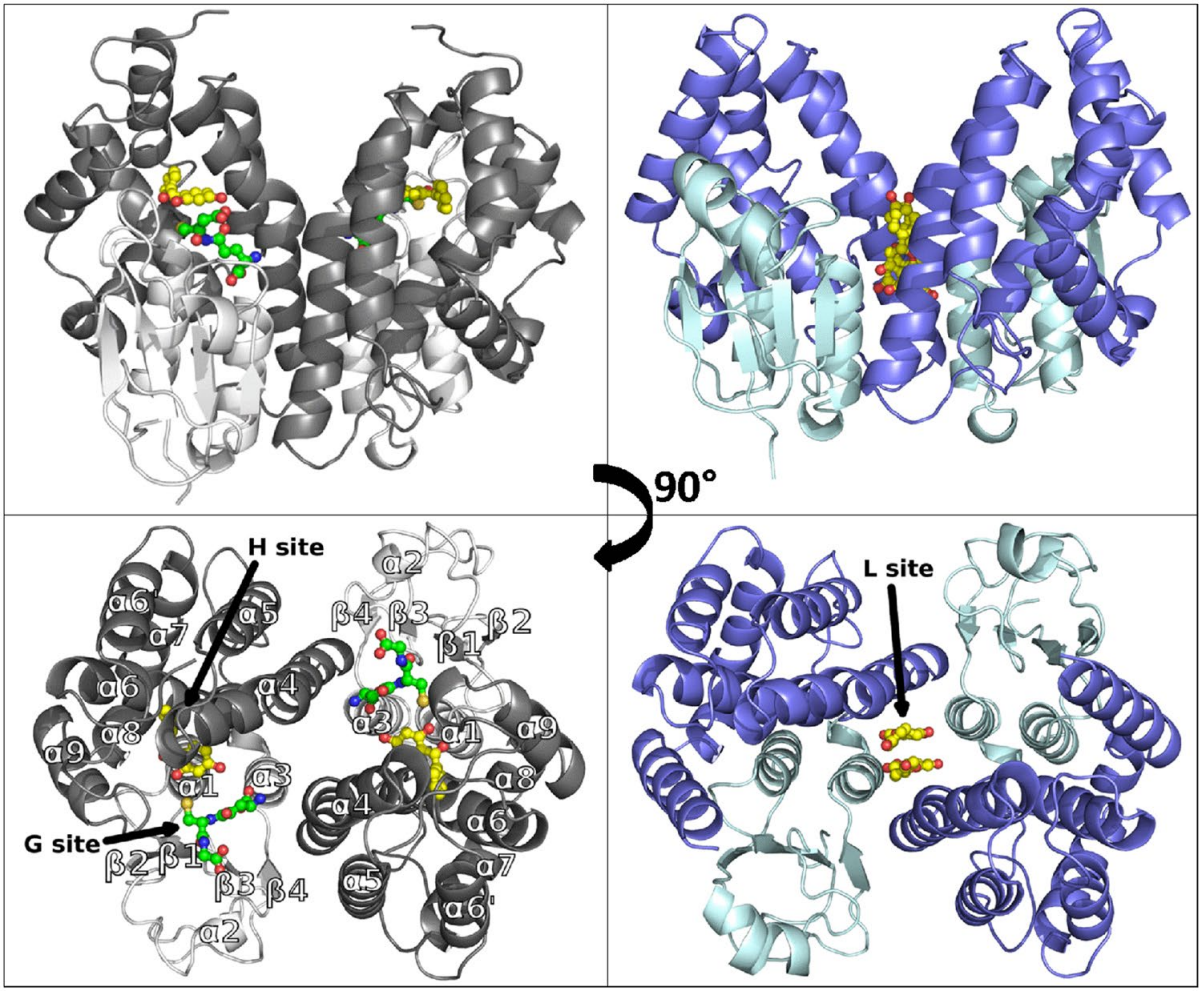
Overall views of *Trametes versicolor* GSTO3S structure in complex with glutathione and 2,4 hydroxy benzophenone (left panels) and GSTO6S structure in complex with naringenin (right panels). In each case, structures are depicted in cartoon mode with ligands shown as spheres and sticks (glutathione in green, 2,4-HBP and naringenin in yellow). N-terminal domains are shown in light colors (white for GSTO3S, cyan for GSTO6S) and C-terminal domains are shown in deeper colors (grey for GSTO3S, blue for GSTO6S). Black arrows indicate positions of glutathione binding site (G-site), hydrophobic binding site (H-site) and ligandin site (L-site). In each case is represented one physiological dimer that typifies the structure of GSTs where the N-terminal domain (secondary structure β_1_α_1_β_2_α_2_β_3_β_4_α_3_) of one monomer cross-interacts with the C-terminal domain (α_4_α_5_α_6_α_6_′α_7_α_8_α_9_) of the second one, and vice-versa.

For further insights into these findings, a structure/function relationship study focused on the analysis of TvGSTO3S-ligand interactions was initiated. The structure of apo TvGSTO3S was determined by X-ray crystallography at a resolution of 1.35 Å (Supplementary Table S1). It has a typical GST fold where the N-terminal thioredoxin domain of one monomer cross-interacts with the C-terminal all-helical domain of the second one, and vice-versa (Fig. 1). TvGSTO3S displays the highest sequence identity with Omega GSTs, though its closest structural homologs identified by PDBeFold^23^ are Tau GSTs. The resemblance between these two classes was already discussed for a wheat Tau GST^24^. However, TvGSTO3S has unique features that distinguish it from previously described GSTs. They mainly include an elongation of the loop between β_3_ and β_4_ and an additional helix α_6_′ (Supplementary Fig. S2).

The N-terminal end of TvGSTO3S helix α_1_ harbors a serine as the catalytic residue instead of the cysteine found in the other GSTOs structurally characterized so far^17,25^. It enables efficient GSH-transferase activity towards usual synthetic substrates and disables reductase activity (Supplementary Table S2). In order to get a detailed picture of the ligand binding sites of TvGSTO3S, we determined the structures of several complexes with free GSH, or some glutathionylated derivatives GS-R (glutathionyl-dinitrobenzene GS-DNB and glutathionyl-phenylacetophenone GS-PAP), or some of the HBPs identified by TSA (2,4-, 3,4-, 2,3,4-, 2,4,4′- HBPs) (Supplementary Table S1). TvGSTO3S harbors a canonical GSH binding site (G-site) made up of polar residues from the N-terminal domain which stabilizes the glutathionyl moiety (GS-) of the tested ligands (Fig. 1, Supplementary Figs S3 and S4). On the contrary, the hydrophobic binding site (H-site) which hosts the -R group of the GS-R ligand has a singular shape relative to most GSTs. While a large open valley is usually observed in a cleft between the two domains^26^, TvGSTO3S exhibits a well-delineated cavity deeply inserted between helices α_4_ and α_6_ of the C-terminal domain. The crystal structures of the different complexes show that this pocket is perfectly suited to accommodate polyaromatic ligands, due to its strong hydrophobic character, while two polar residues are found at the entrance close to the G-site (Supplementary Fig. S3). The phenylacetophenone group of GS-PAP fully fills the cavity, on the contrary to the more polar -R group of GS-DNB that does not enter it and is only slightly stabilized at its entrance, at the interface with the G-site (Supplementary Fig. S4).

**Figure 2:**
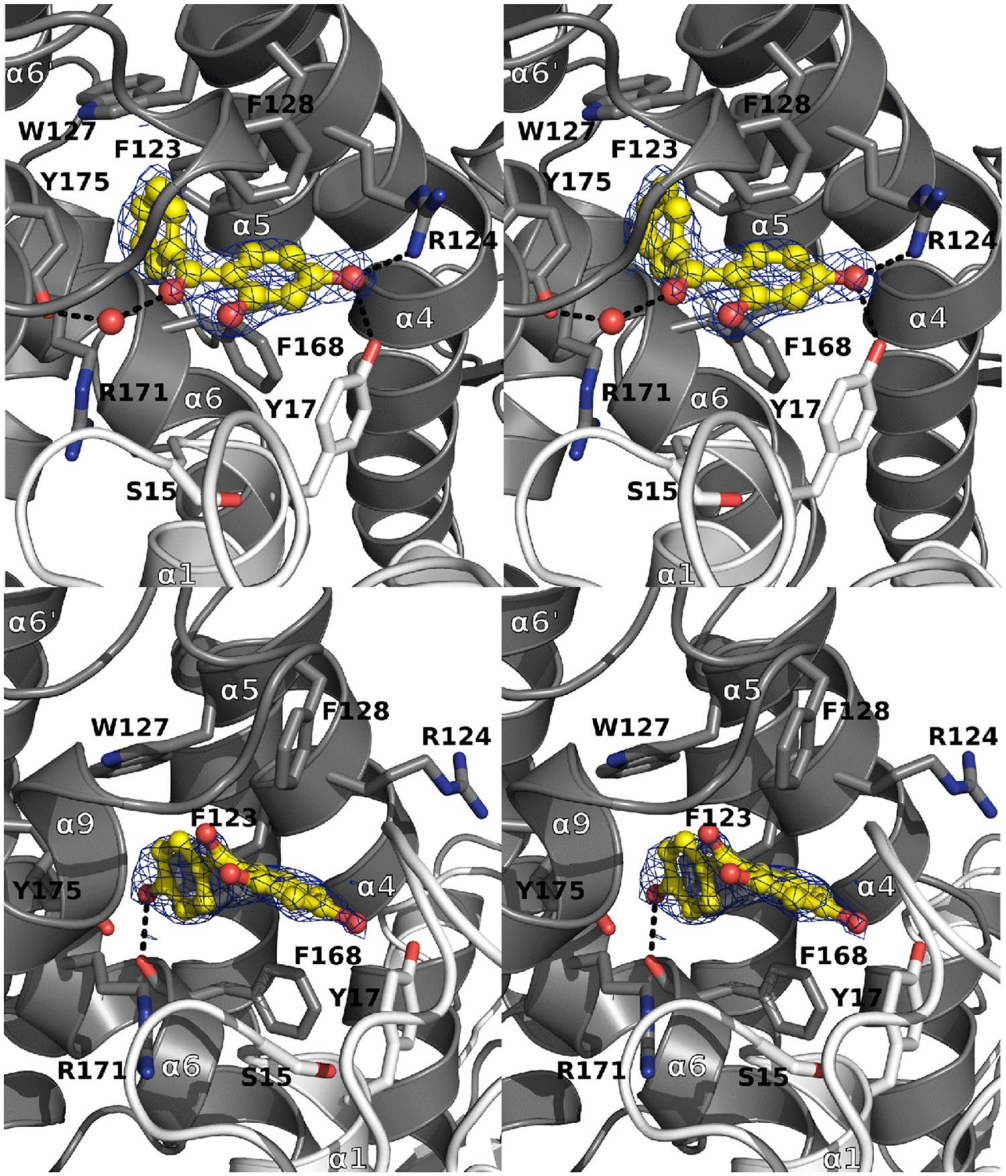
Binding of 2,4- and 2,4,4′-hydroxy benzophenones in GSTO3S hydrophobic binding site. Stereoviews of sections of the GSTO3S complex structures with 2,4-HBP (top view) and 2,4,4′-HBP (bottom view) are shown. TvGSTO3S H-site is a well-delineated cavity deeply inserted in between helices α_4_ and α_6_ of the C-terminal domain. It is perfectly suited to accommodate polyaromatic ligands, due to its strong hydrophobic character given by the aromatic side chains of F123, W127, F128, F168, the aliphatic parts of R171 and Y175, completed with polar residues (Y17 and R124) at the cavity entrance close to the G-site. Polar intermolecular contacts are materialized as dashed lines. Surrounding side chains are represented in sticks. HBPs are shown as yellow sticks and spheres. 2mFo-DFc composite omit maps shown at 1.0 σ around HBPs were calculated by PHENIX.

The four HBPs (2,4-, 2,3,4-, 3,4- and 2,4,4′- HBPs) that were previously identified by TSA also bind in the TvGSTO3S H-site (Fig. 2 and Supplementary Fig. S5). Overall, their phenyl ring B (Table 1) sits deep at the bottom of the pocket and interacts via π-stacking with aromatic amino acid side chains. Their di- or tri-hydroxylated phenyl ring A is closer to the entrance of the cavity and forms hydrogen bonds with polar side chains and water molecules. In more detail, two distinct HBP conformations are observed (*i*) for 2,4-, 2,3,4- and 3,4-HBPs, and (*ii*) for 2,4,4′- HBP, respectively (Fig. 2). Both conformations are common for this family of molecules in the solid state^27^. In (*i*), the central ketone group of HBPs is stabilized via a well conserved water molecule present in all structures, while this interaction no longer exists in (*ii*). For the latter, a new hydrogen bond allows accommodation of the 4′ hydroxyl group at the bottom of the cavity. The presence of this additional 4′ hydroxyl group probably accounts for the distinct conformation (*ii*) observed in the H-site. Finally, another attempt was made to get the structure of a complex between TvGSTO3S and the compound 2,2′, 4,4′-HBP, but no electron density was observed for this putative ligand. These results not only correlate with TSA, but also with the binding affinities of HBPs for TvGSTO3S which were assessed from their capacity to inhibit the GSH-transferase activity towards phenethyl isothiocyanate (PEITC). Indeed, the same four HBPs differentiate from the others in the sense that only their K_i_ values were measurable and found in the μM range (Supplementary Table S3). Altogether, our crystallographic and enzymatic results suggest that the TvGSTO3S H-site is selective for HBPs whose ring B is totally hydrophobic and stabilized at the bottom of the pocket or bears one hydroxyl group in para position whereas ring A is di- or tri- hydroxylated and stabilized at the entrance of the H-site. As shown with TSA, other TvGSTO isoforms also interact with HBPs, each with its own selectivity. The discovery of HBPs as potential ligands for TvGSTOs echoes previous results concerning metabolization of these small molecules by the fungus. For instance, the sunscreen agent BP3 (2-hydroxy, 4-methoxy benzophenone) is metabolized by *T. versicolor* into various HBPs (2,4-, 4,4′- and 4- HBPs) by cytochrome P450 with no oxidation by extracellular laccases^28^ suggesting the importance of intracellular systems to detoxify benzophenones, including phase II enzyme GSTs.

### TvGSTOs interact with flavonoids

In a recent paper, we suggested that GSTs from saprotrophs could interact with flavonoids, which are phenolic compounds as HBPs and present in wood extracts^19^. In order to refine our results, TSA was used to test a set of commercial flavonoids for their interaction with TvGSTOs. Some of the putative complexes were further investigated by X-Ray crystallography. For the first time in GSTs, one structure revealed a symmetrical ligandin site (L-site) filled with a pair of interacting ligands.

As observed for HBPs, TSA shows that each TvGSTO has its own interaction profile with flavonoids and that these interactions are largely related to the number and positions of the hydroxyl groups on the aromatic rings (Supplementary Table S4). While TvGSTO4S and 5S show variable or weak responses to flavonoids, TvGSTO1S, 2S, 3S and 6S mainly show positive shifts of their denaturation temperature. For instance, all molecules but catechin affected TvGSTO6S. Interestingly, these flavonoids were recently detected by mass spectrometry in *T. versicolor* fructifications. Their presence might arise from trees or soils on which the fungi grow^29^.

**Figure 3:**
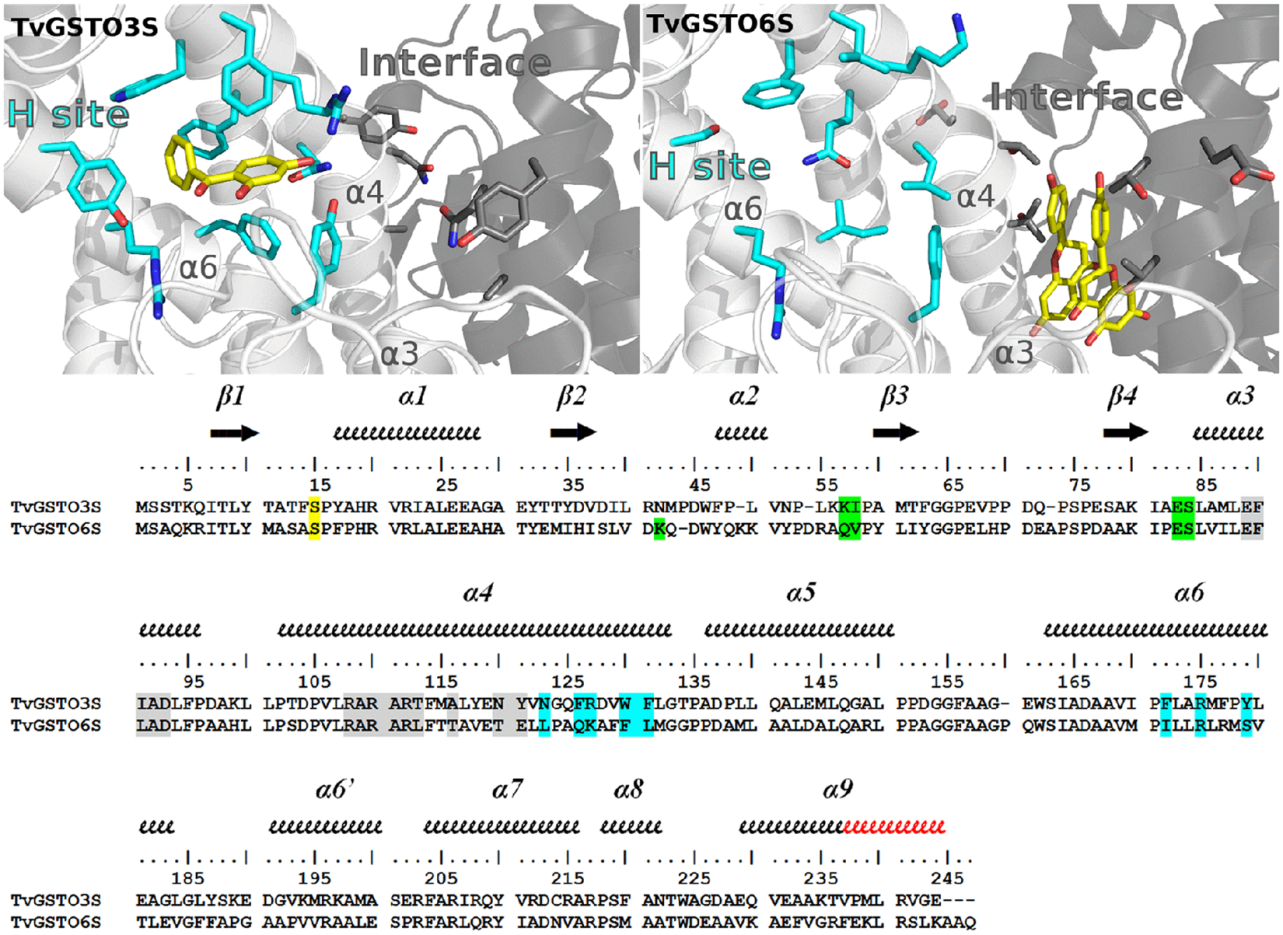
Structural comparison of TvGSTO3S and −6S isoforms showing differences between H-sites and L-sites. The structures shown are TvGSTO3S in complex with 2,4-HBP (top left) and TvGSTO6S in complex with naringenin (top right). In both cases, helix α9 and C-terminal tail were removed from structures for clarity. Monomers A and B are colored white and grey, respectively. 2,4-HBP and naringenin molecules are represented as yellow sticks. H-site and L-sites residues are colored respectively cyan and grey in the structures (highlighted respectively cyan and grey on the structural alignment). G-site residues are highlighted green in the sequences and are not shown on structures for clarity. Catalytic serine is highlighted yellow in the sequences. Secondary structures are labelled and shown using arrows (β-strands) and squiggles (helices). Secondary structure elements are based on TvGSTO6S. Helix α9 extra turns in TvGSTO6S are colored red on the sequence alignment.

The crystal structure of TvGSTO6S was solved at 1.45 Å resolution (Supplementary Table S1). As expected, it reveals the same overall fold than TvGSTO3S (rmsd 0.78 Å, 183 aligned Cα per monomer). While G-sites are made of similar polar residues, differences are observed at the H-site and at the interface between monomers (Fig. 3). The lack of a structure with a ligand that binds in TvGSTO6S H-site precluded its fine description. However, when compared with the equivalent region of TvGSTO3S, significant differences are readily apparent in TvGSTO6S. Most of the residues that line the benzophenone binding site of TvGSTO3S are different. In addition, the extension of the C-terminal α-helix by two extra turns leads to an open and shallow H-site, contrary to the buried H-site in TvGSTO3S covered and closed by the loop corresponding to the extra turns. There are also slight differences between the dimer-interfaces of both isoforms. Most residues at the interface are conserved, except for a few ones situated in helix α4 (Fig. 3). The larger residues of TvGSTO6S (L112 and T115 instead of T110 and A113 in TvGSTO3S) and the loss of the inter-monomer interaction between Y118 (replaced by E120 in TvGSTO6S) and E80 present in TvGSTO3S probably account for a more open dimer in TvGSTO6S. Its interface residues form a pocket centered on the two-fold axis of the dimer (rectangular section of 8 × 6 Å^2^), larger than the equivalent region found in TvGSTO3S (rectangular section of 7 × 4 Å^2^, Supplementary Fig. S6). This region hosts a third binding site named the ligandin site (L-site) in the human GST Omega 1 (hGSTO1)^30^.

**Figure 4:**
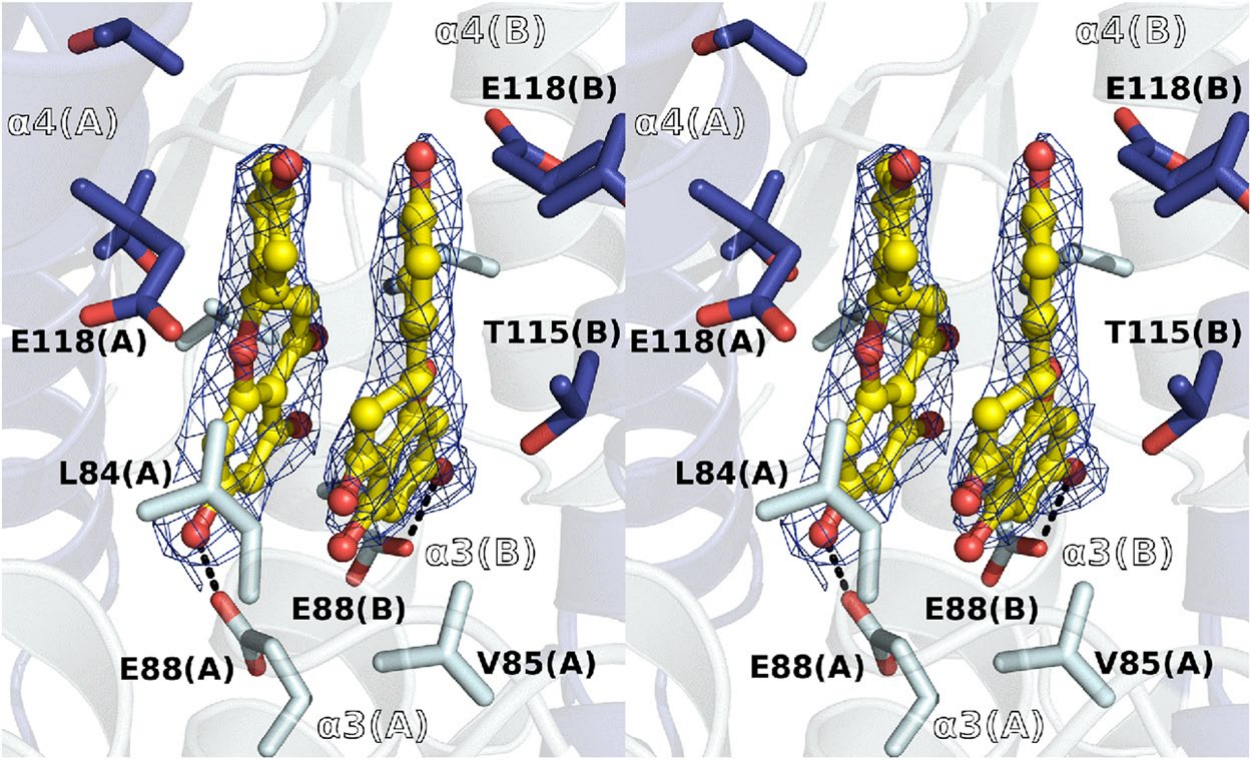
Binding of naringenin in GSTO6S non-catalytic ligandin site. Stereoview of a section of the GSTO6S complex structure with a pair of naringenin molecules is shown. GSTO6S ligandin site is an hydrophobic pocket inserted at the interface of the dimer, between helix α3 and α4 of both monomers around the dimer 2-fold axis. The apolar environment is formed by the aliphatic part of the side chains of both monomers (L84, V85, T115, E118) while a hydrogen bond with E88 stabilizes one of the flavonoid hydroxyl groups. Polar intermolecular contacts are materialized as dashed lines. Surrounding side chains are represented in sticks. Naringenin is shown as yellow sticks and spheres. 2mFo-DFc composite omit map shown at 1.0 σ around the ligand was calculated by PHENIX.

In order to determine the structure of a complex between TvGSTO6S and a flavonoid, several candidate molecules were chosen for soaking experiments. Crystals of TvGSTO6S were unstable and brittle, and required some tricks to prepare complexes. Among the trials performed, one was successful where the droplet that gave rise to TvGSTO6S crystals was deposited for a few hours on a surface previously coated with naringenin (chemical structure in Supplementary Table S4). The structure of the complex was solved at a resolution of 2.3 Å (Figs 1 and 4, Supplementary Table S1). Two naringenin molecules bind in the pocket described above, which constitutes the L-site of TvGSTO6S (Figs 1 and 4). Each naringenin aromatic ring stacks with its symmetrical equivalent in an energetically-favored association (−13.64 kcal/mol calculated by DFT). A pair of stacked flavonoids was already observed bound to dihydroflavonol 4-reductase, however in a head to tail arrangement in the active site of the enzyme, with no dimer symmetry^31^. In TvGSTO6S, the naringenin pair fits the apolar environment formed by the neighboring aliphatic part of the side chains of both monomers (L84, V85, R111, T115, E118 and T119) while a hydrogen bond with E88 stabilizes one of the flavonoid hydroxyl groups. L-sites at the dimer interface were previously described for hGSTO1 (pdb code 4is0)^30^ and for *Arabidopsis thaliana* GST Phi 2 (AtGSTF2, pdb code 5a4v)^32^ however with one ligand only. In hGSTO1, the L-site takes place at a location similar to TvGSTO6S and shows conserved patches of hydrophobic residues along helix α3 (Supplementary Fig. S2). Interestingly, E88 that stabilizes naringenin in TvGSTO6S is conserved in hGSTO1 (E91) where its side chain is found near the nitrophenacyl moiety of the glutathione adduct. The case of AtGSTF2 is quite different. A quercetin molecule sits at the opposite side of the dimer interface, near the C-terminal end of helix α3 and the N-terminal end of helix α4.

Structural comparisons suggest that Omega GSTs have two hydrophobic ligand binding sites: an H-site near α4 and α6 and an L-site at the dimer interface. In the case of TvGSTO6S, the L-site can host flavonoids. The alignment of the solved structures with the sequences of TvGSTO1S, −2S, −4S and −5S shows variations at the putative H- and L-sites (Supplementary Fig. S2), which could explain the isoform-specific TSA patterns.

### Fishing of a flavonoid from a wild-cherry heartwood extract by affinity crystallography

One of the biggest challenges in GST characterization is the identification of natural ligands^33^. Affinity crystallography is a very new method that was recently conceived to select and identify new inhibitors from natural crude extracts as potent drug scaffolds for pharmaceutical targets^34^. We successfully applied this method on TvGSTO3S crystals to isolate the flavonoid dihydrowogonin from a partially purified wild-cherry extract.

**Figure 5:**
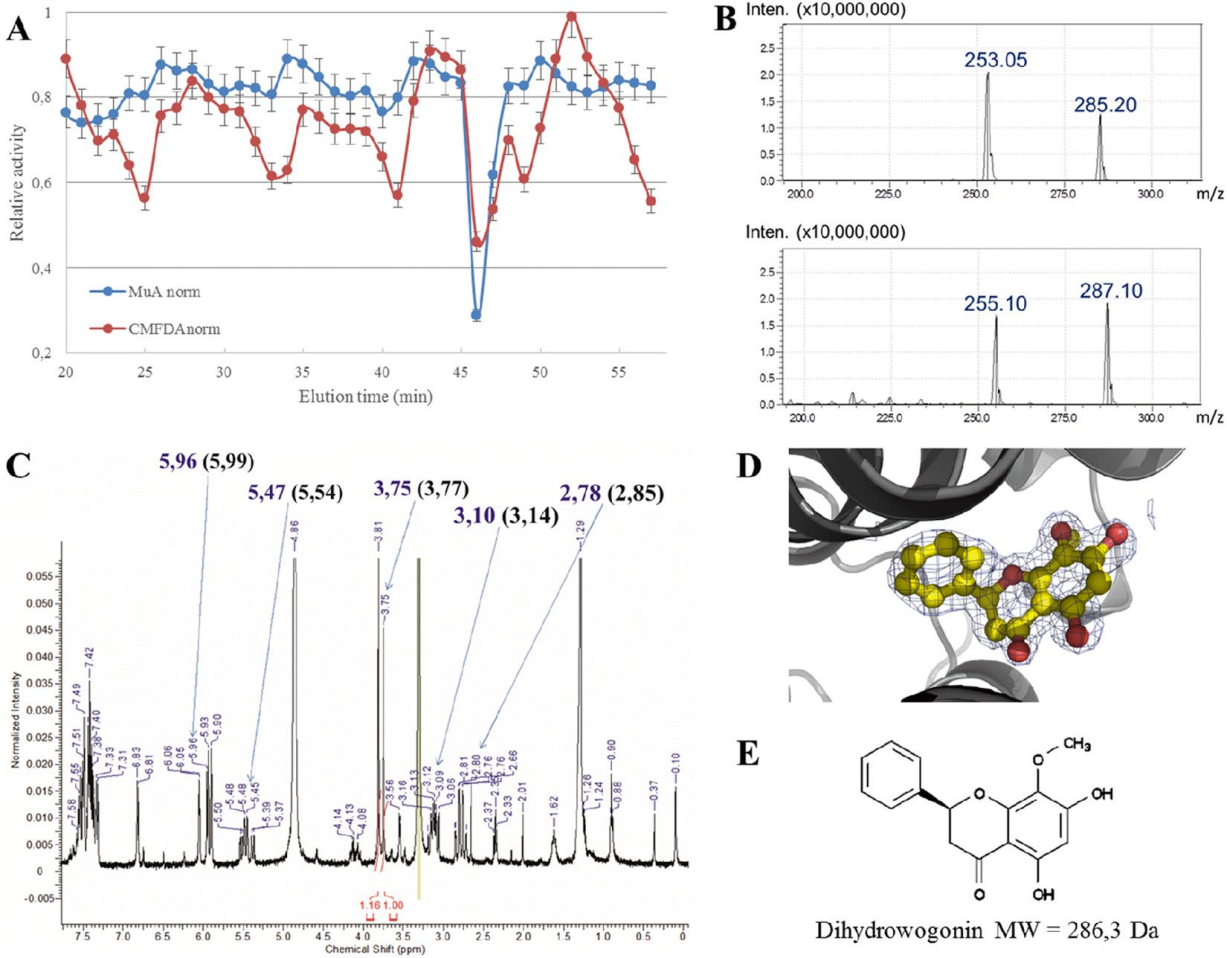
Combined approach including affinity crystallography revealed dihydrowogonin bound to GSTO3S hydrophobic site. (**A**) Normalized inhibition of esterase activity with substrates CMFDA and MUA is shown. Fractions 46–47 min that inhibited both activities were selected for further analysis. (**B**) MS analysis in positive (bottom panel) and negative (top panel) modes revealed two major compounds. (**C**) ^1^H-NMR spectrum showed the structural features of flavanones. Affinity crystallography allowed the elucidation of the flavanone dihydrowogonin. Its ^1^H-NMR data^37^ are indeed found on the spectrum of the mixture: numbers in parenthesis are the typical chemical shifts for dihydrowogonin and numbers in blue correspond to the values obtained in the present study. Integration values of 2 methoxy groups allowed to evaluate maximum abundance of dihydrowogonin in the fractions (numbers in red). (**D**) Electron density of dihydrowogonin in structure of GSTO3S crystallized in presence of 46-47 eluate. The map shown is a 2mFo-DFc composite omit map contoured at 1 σ. (**E**) Chemical structure of dihydrowogonin.

Acetonic extracts of wild-cherry heartwood were fractioned by reverse chromatography. The potential inhibition of the various fractions on TvGSTO esterase activity was analyzed using both chloromethylfluorescein diacetate (CMFDA) and methylumbelliferyl acetate (MUA) as substrates (Fig. 5). In previous studies^13,19^, these substrates had increased detection sensitivity of fluorescence and avoided quenching effects of wood extracts. The fractions that eluted after 46 and 47 min induced a strong inhibition of both measured activities. The pooled mixture was tested for its ability to interact with TvGSTO3S using TSA. A positive 4.8 °C shift of the observed ∆Td confirmed the presence of potential TvGSTO3S ligands. Analysis of this solution by LC-MS revealed the presence of at least two compounds with molecular masses of 254 and 286 g.mol^−1^ respectively (Fig. 5). The 46–47 min eluate also analysed by H^1^NMR (spectrum shown in Fig. 5) exhibited characteristic signals of a flavanone skeleton with, in addition, typical methyloxy group singlets at 3.75 or 3.81 ppm. At this stage, several compounds already described in the extractives composition could correspond to such data (*e.g.* dihydrowogonin or sakuranetin)^35–37^ so that it was impossible to definitively assign the molecular structures, particularly concerning the MeO group position either on the ring A or B (Fig. 5).

Affinity crystallography was then used to further elucidate the interactions between TvGSTO3S and compounds present in the 46–47 min eluate. This approach assumes that a complex can form when the protein is mixed with a partially purified mixture of molecules containing potential ligands. We re-suspended the dried eluate with a minimal volume of DMSO to obtain a concentrated mixture suitable for TvGTO3S crystallization. The addition of 2.5% of the concentrated solution in the mother liquor produced co-crystals as observed from the electron density peak in the H-site. High quality of the signal together with the NMR and MS data led to unambiguous identification of dihydrowogonin (Fig. 5). This flavonoid was previously identified in *Prunus avium* heartwood and described as a flavanone^35^. Interestingly, its corresponding flavone (wogonin) was shown to strongly inhibit the GSH-transferase activity of TvGSTO3S towards PEITC (K_I_ = 2.89 ± 1.24 µM, Supplementary Table S5), while no inhibition was detected for TvGSTO6S. Since in the latter a stabilizing effect is still observed by TSA, the ligand should not bind in its catalytic site (*i.e*. H-site). In TvGSTO3S, the unsubstituted ring of dihydrowogonin sits in the bottom of the H-site like the ring B of HBPs (see above) (Supplementary Fig. S7). The opposite ring points towards the entrance of the cavity the polar residues of which stabilize its methoxy and hydroxyl groups by direct or water-mediated hydrogen bonds. The hydroxyl group situated at position 7 is only 2.6 Å from the glutathione sulfur atom. This short distance questions a possible catalysis with a related substrate. The combined use of X-ray diffraction, NMR and MS succeeded in identifying dihydrowogonin as a natural molecule that originates from wild-cherry heartwood and that tightly binds TvGSTO3S.

## Conclusion

In this study, we demonstrated at the biochemical and structural level that *T. versicolor* GSTs that belong to the Omega class interact with polyphenolic compounds found in wood, and in particular with flavonoids such as dihydrowogonin and naringenin. Indeed, white-rot fungi such as *T. versicolor* have to cope with potentially toxic tree secondary-metabolites mainly constituted of polyphenolic compounds, which accumulate in different parts of the wood (heartwood, knots). The molecular interactions between TvGSTOs and polyphenols appear to be very diverse. They potentially involve two structural sites for each isoform. It is tempting to establish a correlation between the diversity of these interactions and the extension of the Omega class (at least 16 isoforms) in *T. versicolor*, which encounters a large diversity of polyphenols in its natural environment. However, the exact function of this GST network remains unclear since no catalytic activity against the tested polyphenols has been detected. The ability of TvGSTOs to bind polyphenols through different sites suggests that fungal GSTs could be involved in the transport of various polyphenols. In plants, GSTs were shown to facilitate the flavonoid transport from the cytoplasm into the vacuole^38^. These GSTs could act as flavonoid and glutathione carriers, providing ABC transporters with both molecules for their co-transport. This transport requires free glutathione but no glutathionylation activity^39^. Our study suggests that fungal GSTs as their plant homologues could be involved in the transport and sequestration of flavonoids.

## Methods

### Reagents

All pure molecules together with the fluorescent marker SYPRO® Orange used in TSA were purchased from Sigma-Aldrich (St. Louis, MO, USA), except for wogonin provided by Extrasynthese (Genay, France).

### Production and purification of proteins

The production in *E. coli* (Rosetta2 DE3 pLysS strain, Novagen) and purification of the six selected TvGSTOs (accession number in the JGI database: TvGSTO1S: Tv75639; TvGSTO2S: Tv56280; TvGSTO3S: Tv48691; TvGSTO4S: Tv65402; TvGSTO5S: Tv54358 and TvGSTO6S: Tv23671) were performed as explained previously^19^.

### Study of the thermostability of TvGSTOs

The experiments were performed in 96 well microplates (Harshell, Biorad) and the measurements carried out using a real time PCR detection system (CFX 96 touch, Biorad). The assays were performed as follows: 5 μL of Tris-HCl (150 mM) pH 8 buffer, 2 μL of pure molecules dissolved in DMSO (final concentration: 0.8 mg/mL for molecules of the chemical library; 100 µM for benzophenones and flavonoids), 5 μL of proteins (contained in Tris-HCl 150 mM, pH 8; final concentration: 40 µM for the tests with the chemical library; 10 μM for the other tests), 2 μL of SYPRO orange (previously diluted 80−fold in ultra-pure water) and 11 μL of ultra-pure water for a total volume of 25 μL per well. The plate was centrifuged 30 seconds at 4000 g. Fluorescence was measured (excitation: 485 nm; emission: 530 nm) every minute starting from 3 minutes at 5 °C while increasing temperature from 5 to 95 °C with a step of 1 °C per minute. The denaturation temperature (Td), which corresponds to the temperature where 50% of the total fluorescence is measured, was determined by using the non-linear regression Boltzmann sigmoidal model in GraphPad Prism 6 software for data obtained in presence of potential ligands, while the reference was considered for similar experiments conducted by adding DMSO only.

### Inhibition Kinetics

Competition tests between phenethyl isothiocyanate (PEITC) and benzophenones or flavonoids were performed in a final volume of 500 µL. Inhibition tests of the glutathionylation activity were assayed with various concentrations of PEITC (25–300 µM) and a fixed concentration of GSH (1 mM) in presence or not of inhibitors. These experiments were carried out in 100 mM pH 6.4 phosphate buffer at 25 °C by analyzing the glutathionylated product which absorbs at 274 nm. Basal activity of samples containing GSH, PEITC and benzophenones was subtracted from the enzyme catalyzed rates. The inhibition constants (Ki) were calculated using the GraphPad software with the nonlinear regression based on the mixed model inhibition.

Inhibition tests of the esterase activity of TvGSTO3S with CMFDA or MUA as substrates in presence of extract fractions were performed in 96-well microplates. The emission of fluorescence at 517 nm and 460 nm after an excitation at 492 nm and at 355 nm for CMFDA and 4-MUA respectively was followed using microplate reader (2030 Multilabel Reader Victor X5, PerkinElmer). Reactions were performed in 200 µL of Tris-HCl pH 8 (30 mM), EDTA (1 mM) buffer, with 0.5 µM of CMFDA, 5 µM of 4-MUA and 1 mM of GSH. Using both fluorescent substrates, TvGSTO3S activity was measured in presence or in absence of 2 µL of the tested fractions. The ratio between the two slopes (∆RFU/min, Relative Fluorescence Unit) has been used to determine the potential inhibition.

### Fractionation of wild-cherry hardwood acetonic extracts by HPLC

Wild-cherry hardwood acetonic extracts were fractioned by high-performance liquid chromatography. 100 µL of extract at 10 mg/mL were fractioned by reverse chromatography using Kinetex biphenyl column (250 × 4,6 mm) previously equilibrated with H_2_O/Formic acid 0.1% buffer. The molecules adsorbed to the column were eluted with the help of a gradient of methanol (from 0 to 100%). Collected fractions were evaporated using SpeedVac™ (UniEquip) and finally dissolved in DMSO.

### Crystallogenesis experiments

Crystallization of TvGSTO3S was assayed by the microbatch under oil method at 278 K. TvGSTO3S (13 mg/mL) crystallized by mixing 1 µL of protein with 3 µL of commercial solution consisting in 30% (w/v) PEG 400, 0.2 M calcium acetate in 0.1 M pH 4.5 acetate buffer (Wizard Classic Screen 1, Rigaku). Crystals of the complexes of TvGSTO3S with glutathione (TvGSTO3S - GSH), TvGSTO3S with glutathionyl-dinitrobenzene (TvGSTO3S - GS-DNB), TvGSTO3S with hexyl-glutathione (TvGSTO3S - GS-hexyl) and TvGSTO3S with glutathionyl-phenylacetophenone (TvGSTO3S - GS-PAP) were obtained by soaking apo TvGSTO3S crystals during one hour in the mother liquor containing 10 mM ligand (0.5 mM in the case of GS-PAP). Crystals of the complexes of TvGSTO3S with HBPs (2,3,4-trihydroxybenzophenone, 3,4-dihydroxybenzophenone, 2,4-dihydroxybenzophenone and 2,4,4′-trihydroxybenzophenone) were obtained by co-crystallizing the protein pre-incubated for 30 min with 10 mM ligand. Complex of TvGSTO3S with dihydrowogonin was obtained in a similar way by using 10 mg/mL of partially-purified wild-cherry hardwood extract.

First screening of the TvGSTO6S crystallization conditions was performed by using an Oryx 8 robot (Douglas Instruments) to implement sitting drops with commercial kits of various solutions. Crystals were then optimized by the hanging drop method. The droplet was prepared by mixing 1 µL of TvGSTO6S (26 mg/mL) with 0.2 µL of a crystal seed stock obtained by crushing the droplet content of the best hits of the screening step and with 1 µL of a crystallization solution consisting in 25% (w/v) PEG 1500, 0.1 M pH 6.5 MMT buffer (containing DL-malic acid, MES and Tris base in the molar ratios 1:2:2, respectively). The reservoir contained 1 mL of the same crystallization condition. Attempts using classical co-crystallization or soaking experiments failed to prepare crystals of TvGSTO6S complexes with flavonoids. A new strategy was developed based on the dry co-crystallization method^40^. In this original ‘dry soaking’ technique, 0.2 µL of naringenin (100 mM) solubilized in DMSO was deposited on a cover slide and left to complete evaporation. Then, one TvGSTO6S crystal together with 1 µL of its mother liquor was dispensed on the dried naringenin allowing partial ligand resolubilization. The cover slide was then reinstalled above the reservoir that initially allowed crystallization until crystal harvest (ca. 1 day).

### Data collection, processing and refinement

TvGSTO3S and −6S crystals were flash-frozen after a quick soaking in their mother liquor complemented with 20% (v/v) glycerol as cryoprotectant. Primary X-ray diffraction experiments were carried out in-house on a laboratory diffractometer (Agilent SuperNova with CCD detector). Data collection up to 2.5 Å resolution allowed preliminary analysis, especially for ligand screening in TvGSTO3S active site. High resolution diffraction experiments were carried out on the ESRF beamlines FIP BM30A and ID30B (Grenoble, France). TvGSTO3S and −6S native crystals diffracted up to 1.35 Å and 1.48 Å, respectively. Data sets were indexed, integrated and scaled with XDS^41^. The structure of TvGSTO3S was solved by molecular replacement using MR BUMP automated pipeline from CCP4 suite^42^ with the coordinates of poplar GST Lambda 3 (PDB code 4PQI) as the search model. The electron density of a small molecule that we failed to identify was observed in the H-site. It probably bound to the enzyme during the purification process and was not modelled in the electron density. Some of the structures of TvGSTO3S in complex with ligands displayed electron density corresponding to glutathione remaining from the purification process. When present, this residual GSH was modelled, sometimes with partial occupancy. The structure of TvGSTO6S was solved by molecular replacement using PHASER with the coordinates of TvGSTO3S. All structures were refined with PHENIX^43^ and built with COOT^44^. Restraint files for ligands were generated with phenix.elbow and the grade server (URL http://grade.globalphasing.org/cgi-bin/grade/server.cgi). In all of the concerned structures, the occupancies of the ligands added by co-crystallization or soaking techniques were set to 1 and the corresponding B factors were compatible with full presence of the molecules in their binding sites (except for 2,3,4-HPB where the occupancy is 0.8). Validation of all structures was performed with MolProbity^45^ and the wwPDB validation service (http://validate.wwpdb.org). Coordinates and structure factors have been deposited in the Protein Data Bank. Data-collection and refinement statistics of all structures are shown in Supplementary Table S1. Stereo images of a portion of 2mFo-DFc electron density maps are shown in Supplementary Table S6 to assess quality of the structural data. All figures were prepared by using Pymol (The PyMOL Molecular Graphics System, Version 2.0 Schrödinger, LLC).

### Density functional theory (DFT) calculation of naringenin dimer stabilization in TvGSTO6S (Gaussian09 software)

The molecular structure of the dimer of naringenin molecules was extracted from the experimental X-ray structure of TvGSTO6S. Geometry optimization of the hydrogen atoms alone was then performed^46^ at the DFT level of theory in vacuum (*i.e*. no environment effect were taken into account), employing the B3LYP functional^47^ completed with Grimme’s D3 dispersion correction^48^; the 6–111 G(d,p) basis set was used, and basis set superposition errors were corrected by the counterpoise method of Bernardi^49^. The complexation energy at the converged geometrical structure was −13.64 kcal/mol, indicating a strong stabilization of the naringenin molecules within their dimer as found in TvGSTO6S.

### Data Availability

The atomic coordinates of the crystal structures from this publication have been deposited to the Protein Data Bank (https://www.rcsb.org) and assigned the PDB codes 6F43, 6F4B, 6F4F, 6F4K, 6F51, 6F66, 6F67, 6F68, 6F69, 6F6A, 6F70 and 6F71.

### Accession codes

Accession numbers of TvGSTOs in the JGI database are as follows: TvGSTO1S: Tv75639; TvGSTO2S: Tv56280; TvGSTO3S: Tv48691; TvGSTO4S: Tv65402; TvGSTO5S: Tv54358 and TvGSTO6S: Tv23671.

## Electronic supplementary material

supplementary information
