# Supplementary material for: Molecular recognition of wood polyphenols by phase II detoxification enzymes of the white rot Trametes versicolor
Source: Sci Rep. 2018 May 31;8:8472. doi: 10.1038/s41598-018-26601-3 (PMC5981210; doi:10.1038/s41598-018-26601-3)
Supplement: Supplementary file 1 — supplementary information [file 41598_2018_26601_MOESM1_ESM.docx]

**Supplementary Information**

Title: Molecular recognition of wood polyphenols by phase II detoxification enzymes of the white rot *Trametes versicolor*

Mathieu Schwartz^1†^, Thomas Perrot^2†^, Emmanuel Aubert^1^, Stéphane Dumarçay^3^, Frédérique Favier^1^, Philippe Gérardin^3^, Mélanie Morel-Rouhier^2^, Guillermo Mulliert^1^, Fanny Saiag^2^, Claude Didierjean^1*^ and Eric Gelhaye^2*^

1 Université de Lorraine, CNRS, CRM2, Nancy, France

2 Université de Lorraine, INRA, IAM, Nancy, France.

3 Université de Lorraine, LERMAB, Nancy, France

*Corresponding authors: claude.didierjean@univ-lorraine.fr; eric.gelhaye@univ-lorraine.fr

†These authors contributed equally to this work.

**Table of content**

**Supplementary Figures**

[Figure S1. Effects of different compounds from the chemical library on the thermostability of six TvGSTOS isoforms 5](#_Toc509567878)

[Figure S2. Multiple sequence and structure alignment of GSTs from *T. versicolor*, human GSTO1 and wheat GSTU 6](#_Toc509567879)

[Figure S3. GSTO3S active site composed of the glutathione binding site (G-site) and the hydrophobic binding site (H-site) 7](#_Toc509567880)

[Figure S4. Binding of glutathionyl-derivatives in the GSTO3S active site 8](#_Toc509567881)

[Figure S5. Binding of 3,4- and 2,3,4-hydroxy benzophenones in the GSTO3S hydrophobic binding site (H-site) 9](#_Toc509567882)

[Figure S6. Slice view of the L-sites of TvGSTO3S (left) and TvGSTO6S (right) located at the dimer interface 10](#_Toc509567883)

[Figure S7. Binding of dihydrowogonin in the GSTO3S hydrophobic binding site (H-site) 11](#_Toc509567884)

**Supplementary Tables**

[Table S1. Diffraction and refinement statistics 12](#_Toc509567885)

[Table S2. Kinetic parameters of TvGSTO3S and TvGSTO6S toward CDNB (1-chloro-2,4-dinitrobenzene), PEITC (phenethyl isothiocyanate) and GS-PAP (glutathionyl phenylacetophenone) 16](#_Toc509567886)

[Table S3. Summary of the results obtained with thermal-shift assays and inhibition constants (Ki) obtained for TvGSTOSs with hydroxybenzophenones (HBPs) 17](#_Toc509567887)

[Table S4. Effects of several flavonoids on the thermostability of TvGSTOSs 18](#_Toc509567888)

[Table S5. Inhibition constants (Ki) of TvGSTO3S and TvGSTO6S by wogonin and naringenin 19](#_Toc509567889)

[Table S6. Stereo images of 2mFo-DFc electron density maps for each of the eleven structures solved in this study 20](#_Toc509567890)

[**References** 22](#_Toc509567891)


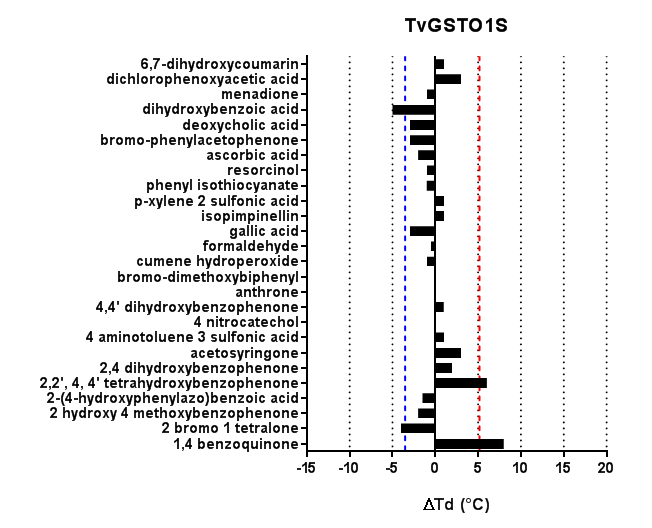


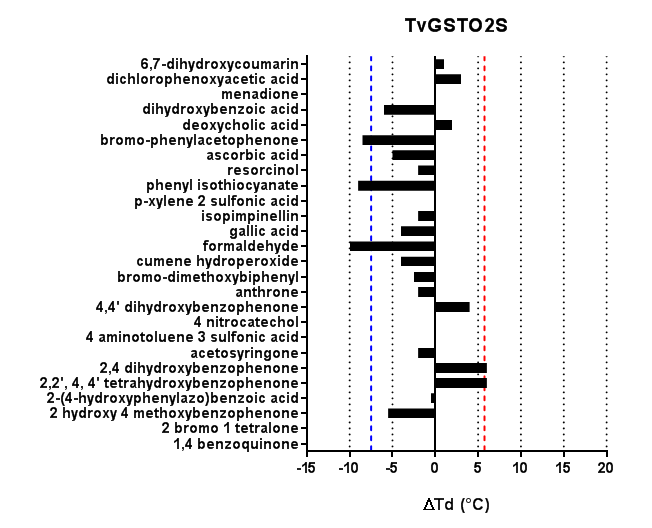


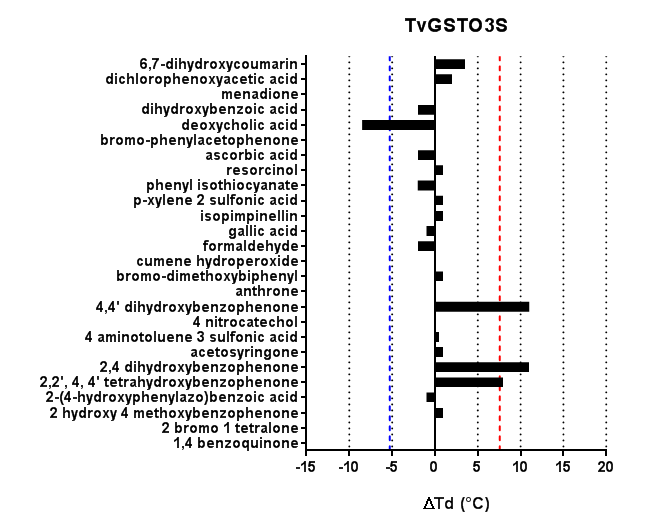


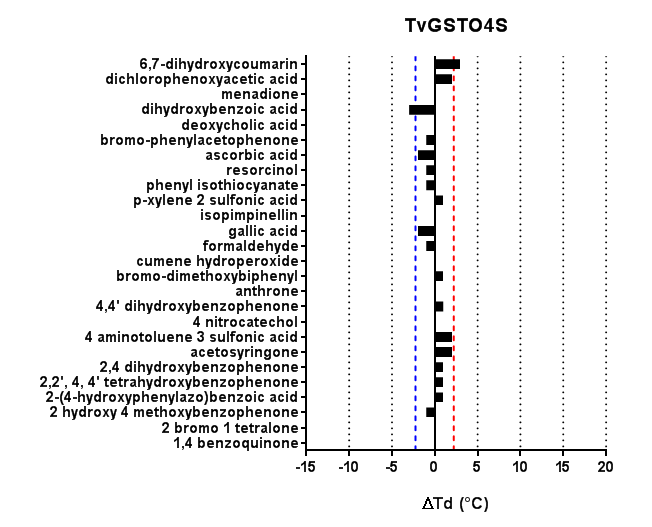


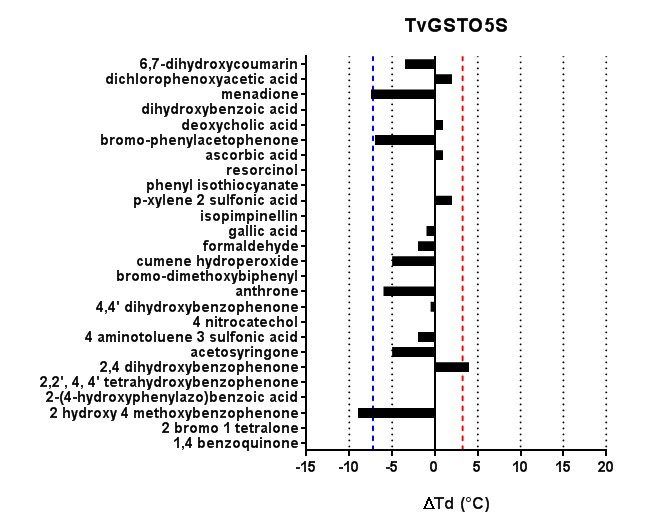


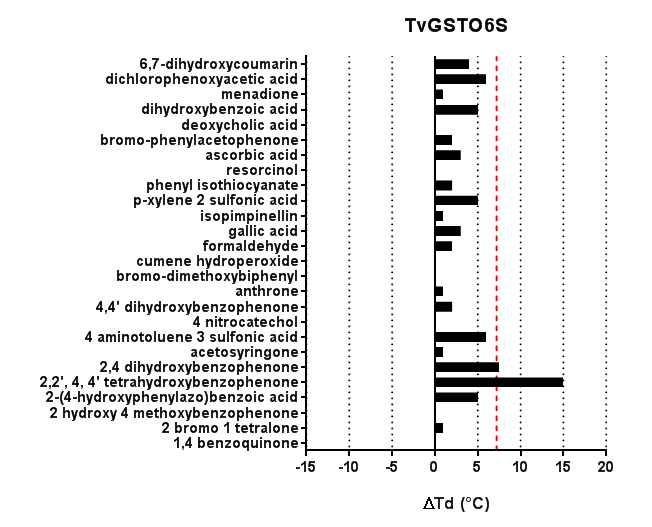


Figure S1. Effects of different compounds from the chemical library on the thermostability of six TvGSTOS isoforms

Red and blue lines correspond to the addition of the average with the positive and negative standard deviations respectively. Thermostability has been analysed by using 40 µM of protein; the final concentration of molecules tested was of 0.8 mg/mL in each well.

**

**

# Figure S2. Multiple sequence and structure alignment of GSTs from *T. versicolor*, human GSTO1 and wheat GSTU

Sequences of TvGSTOs were retrieved from the Joint Genome Institute (URL https://jgi.doe.gov/) database (accession numbers: TvGSTO1S 75639, TvGSTO2S 56280, TvGSTO3S 48691, TvGSTO4S 65402, TvGSTO5S 54358 and TvGSTO6S 23671). Structures of TvGSTO3S and TvGSTO6S solved in this study and structures of human GSTO1 (PDB code 1eem) and wheat AtGSTU (PDB code 1gwc) were used as input structures in Promals3D [^1^](#_ENREF_1). Catalytic motifs, conserved glutathione binding site residues and conserved hydrophobic binding site residues of TvGSTOS isoforms are highlighted in yellow, green and blue, respectively. Residues of the non-catalytic ligandin site of TvGSTO6S and corresponding positions in TvGSTOS isoforms are highlighted in grey. Secondary structures identified in TvGSTO3S structure are reported above the alignment. Residues are numbered according to the TvGSTO3S sequence.

**
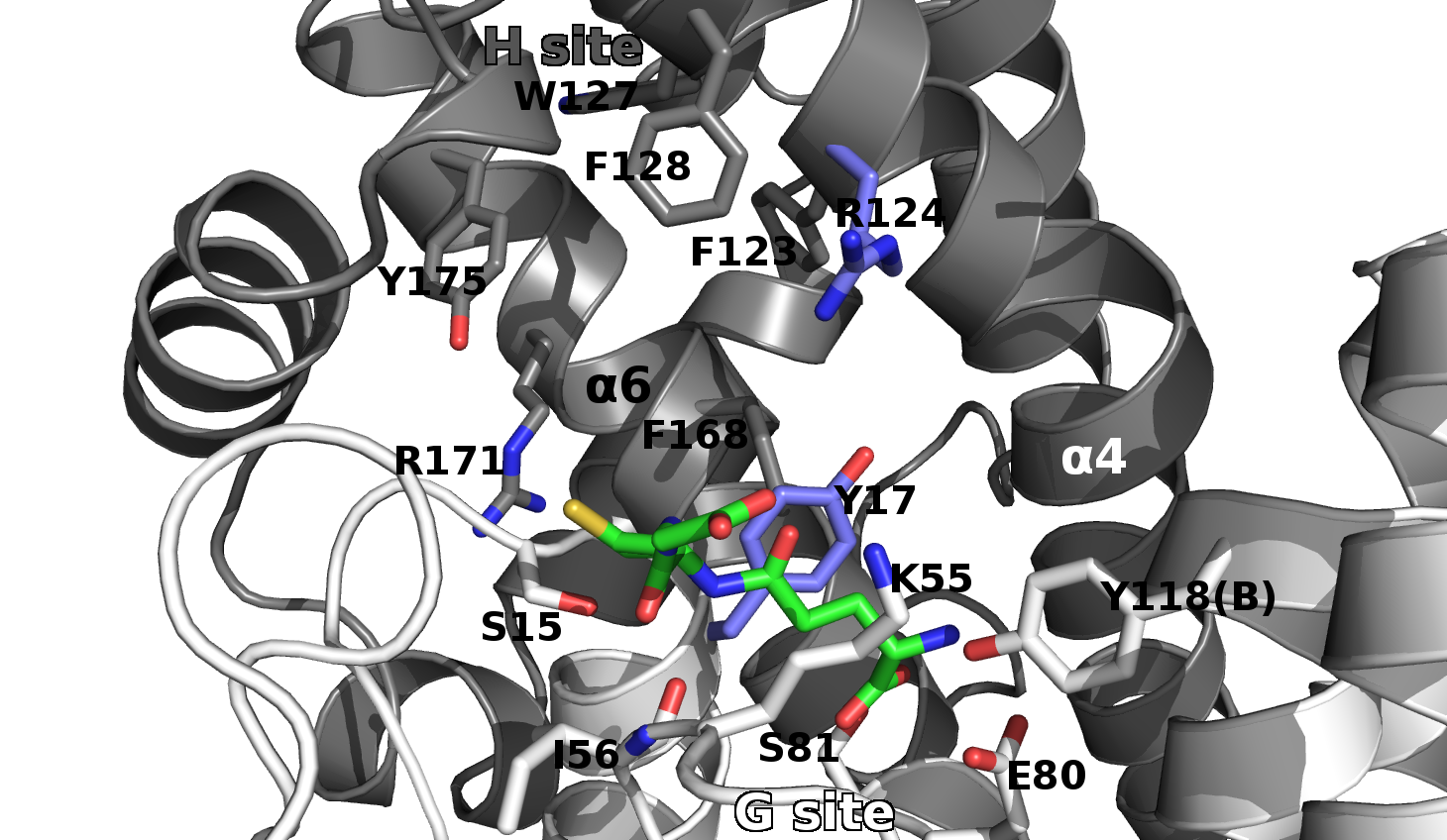
**

Figure S3. GSTO3S active site composed of the glutathione binding site (G-site) and the hydrophobic binding site (H-site)

Section of the TvGSTO3S active site is shown. N-terminal domain bearing the G-site residues is displayed in white and C-terminal domain bearing the H-site residues is shown in black. Glutathione is represented as green sticks. Polar residues at the entrance of the H-site are colored in violet.

| **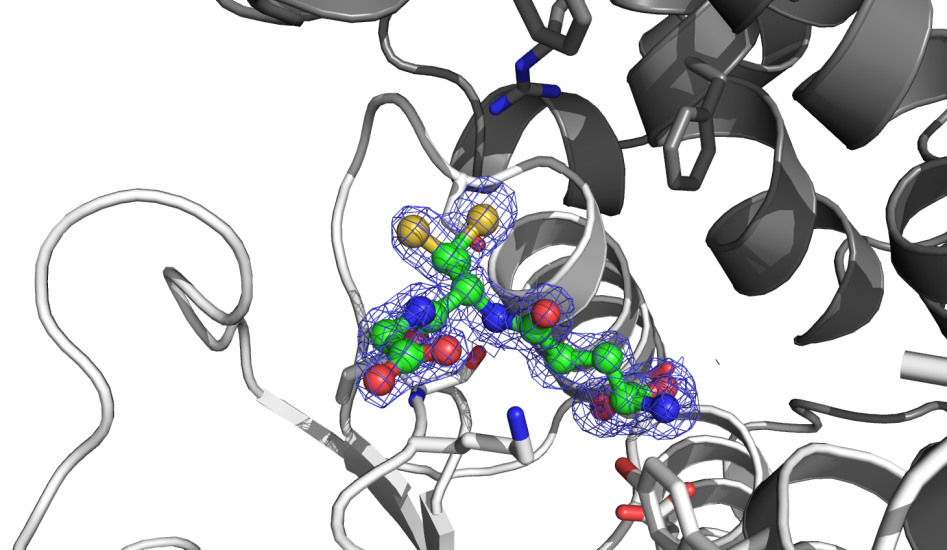** | **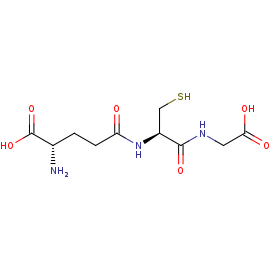**  **Glutathione (GSH)** |
| --- | --- |
| **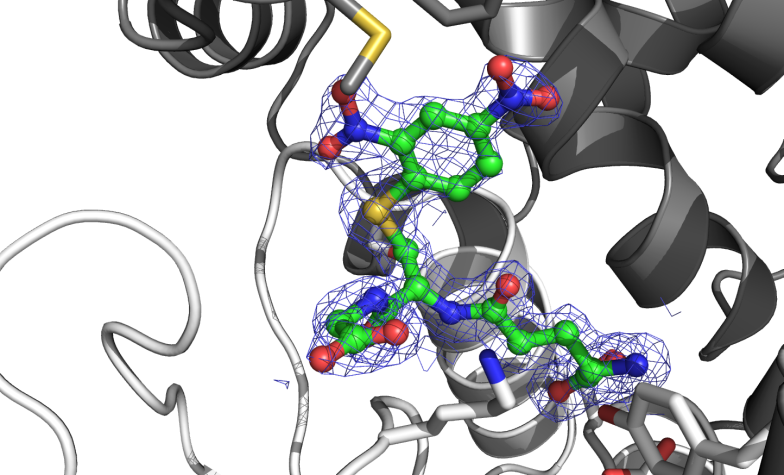** | **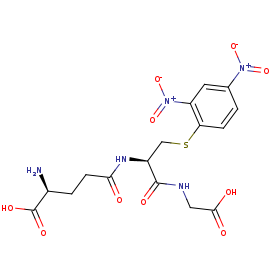**  **Glutathionyl dinitrobenzene (GS-DNB)** |
| **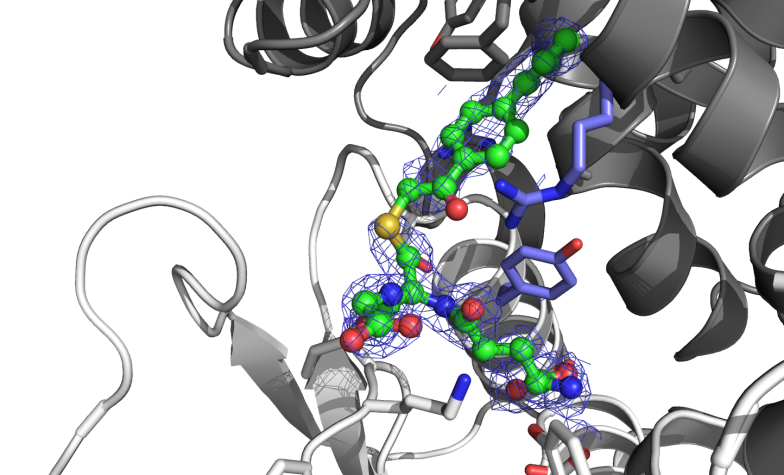** | **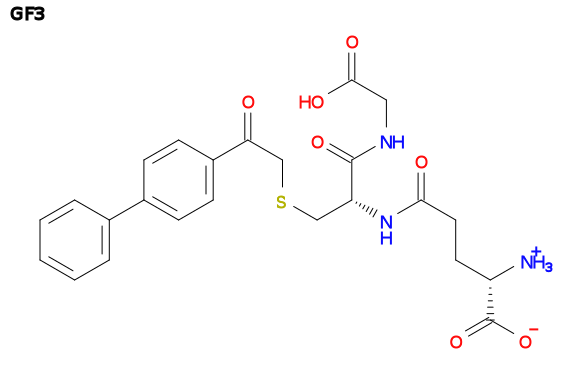**  **Glutathionyl phenylacetophenone (GS-PAP)** |

Figure S4. Binding of glutathionyl-derivatives in TvGSTO3S active site

Sections of TvGSTO3S complex structures with glutathionyl-derivatives are shown in the left column. The corresponding chemical structures of the glutathionyl-derivatives are shown in the right column. Surrounding side chains are represented by sticks. Glutathionyl-derivatives are shown as green sticks and spheres. 2mFo-DFc composite omit maps shown at 1.0 σ around glutathionyl-derivatives were calculated by PHENIX.

**

**

Figure S5. Binding of 3,4- and 2,3,4-hydroxy benzophenones in the GSTO3S hydrophobic binding site (H-site)

Stereoviews of sections of the TvGSTO3S complex structures with 3,4-HBP (top view) and 2,3,4-HBP (bottom view) are shown. Polar intermolecular contacts are materialized as dashed lines. Surrounding side chains are represented by sticks. HBPs are shown as yellow sticks and spheres. 2mFo-DFc composite omit maps shown at 1.0 σ around HBPs were calculated by PHENIX.

| 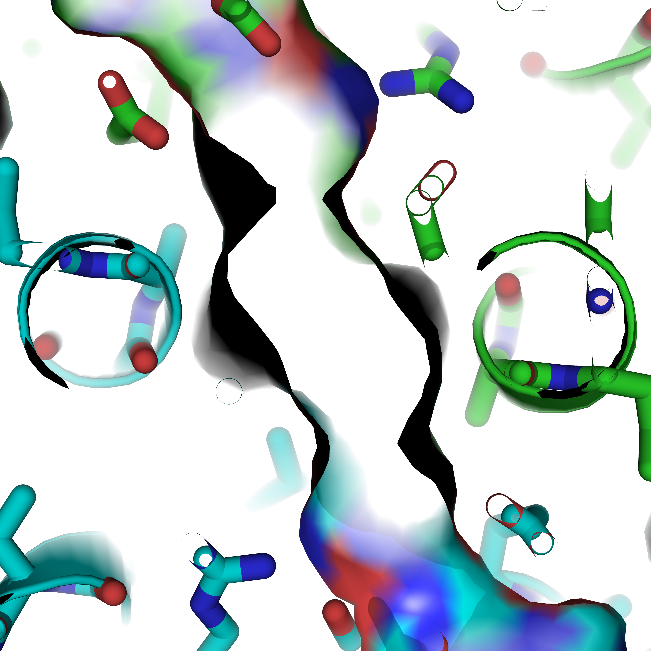 7*4 Å^2^  **Monomer A**  **Monomer B**  **α4**  **α6**  **α6**  **α4** | 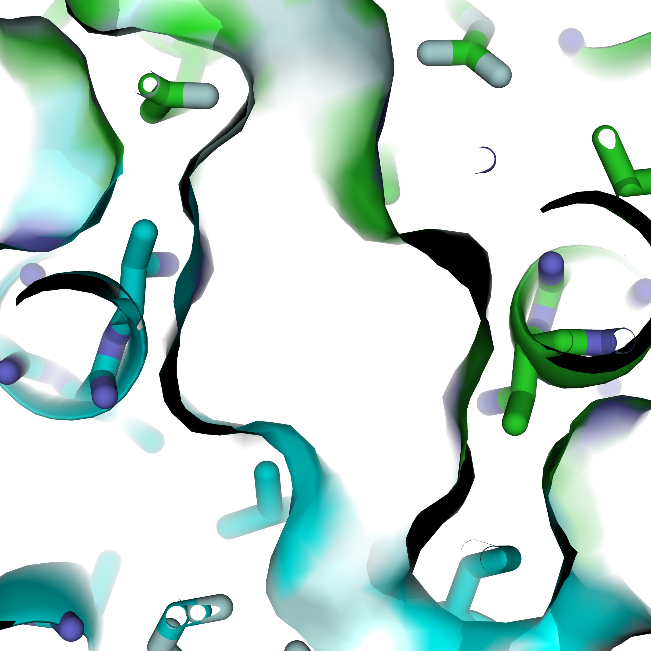 8*6 Å^2^  **Monomer A**  **Monomer B**  **α4**  **α6**  **α6**  **α4** |
| --- | --- |

# Figure S6. Slice view of the L-sites of TvGSTO3S (left) and TvGSTO6S (right) located at the dimer interface

The monomers A (cyan) and B (green) are related by a two-fold axis depicted as a black ellipsoid. Both L-sites exhibit a rectangular section with dimensions 7 Å by 4 Å for TvGSTO3S and 8 Å by 6 Å for TvGSTO6S. The protein surface is represented by a Connolly surface generated with PyMol.


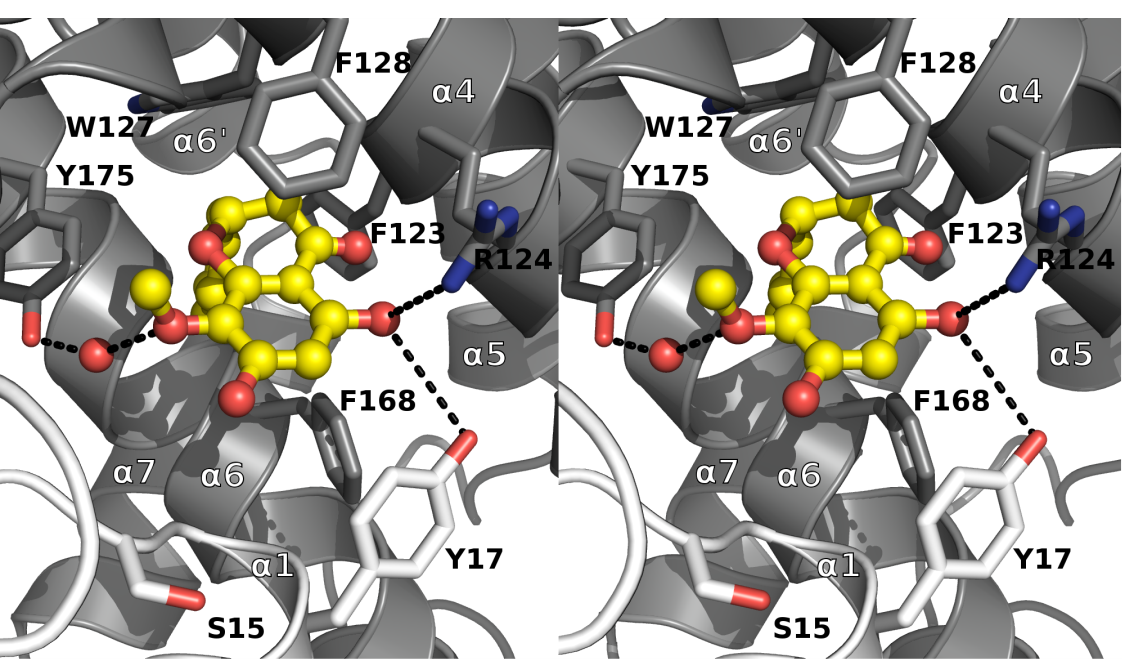


Figure S7. Binding of dihydrowogonin in the GSTO3S hydrophobic binding site (H-site)

Stereo view of the TvGSTO3S complex structure with dihydrowogonin is shown. Polar intermolecular contacts are materialized as dashed lines. Surrounding side chains are represented in sticks. Dihydrowogonin is shown as yellow sticks and spheres.

Table S1. Diffraction and refinement statistics

R_merge_ = $\sum_{hkl} \sum_{i} \left| I_{i}\left( hkl \right)- \left\langle I\left( hkl \right) \right\rangle\right|/\sum_{hkl} \sum_{i} I_{i}\left( hkl \right)$.

R_meas_ = $\sum_{hkl} \left\{ N\left( hkl \right)/\left[ N\left( hkl \right)-1 \right] \right\}^{1/2} \sum_{i} \left| I_{i}\left( hkl \right)-\left\langle I\left( hkl \right) \right\rangle\right|/\sum_{hkl} \sum_{i} I_{i}\left( hkl \right)$.

CC_1/2_ is the correlation coefficient of the mean intensities between two random half-sets of data [^2^](#_ENREF_2).

R_work_ = $\sum_{hkl} \left| \left| F_{obs} \right|-\left| F_{calc} \right| \right|/\sum_{hkl} \left| F_{obs} \right|$. 5 % of reflections were selected for R_free_ calculation. R.m.s.z. : root mean square Z-score [^3^](#_ENREF_3). The molprobity clashscore is the number of serious clashes per 1000 atoms [^4^](#_ENREF_4). The molprobity score is a log-weighted combination of the clashscore, percentage Ramachandran not favoured and percentage bad side-chain rotamers [^4^](#_ENREF_4). Values in parentheses are for highest resolution shell.

Table S2. Kinetic parameters of TvGSTO3S and TvGSTO6S toward CDNB (1-chloro-2,4-dinitrobenzene), PEITC (phenethyl isothiocyanate) and GS-PAP (glutathionyl phenylacetophenone)

The kinetic parameters were calculated using the GraphPad software with the nonlinear regression based on the Michaelis-Menten. “ND” means that no activity has been detected.


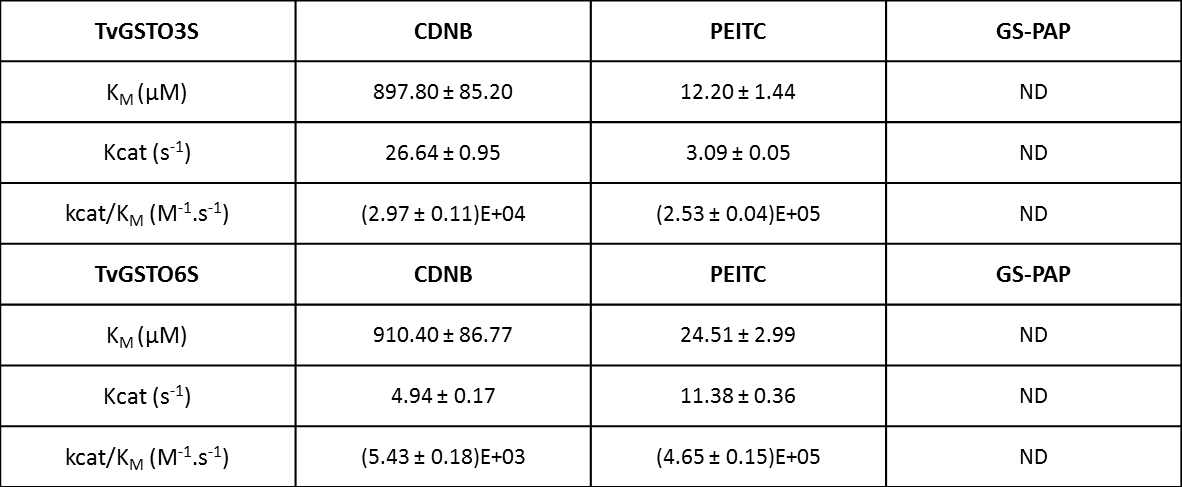


Table S3. Summary of the results obtained with thermal-shift assays and inhibition constants (Ki) obtained for TvGSTOSs with hydroxybenzophenones (HBPs)

“NS” means that temperature denaturation has not been significantly modified. “∆Td” corresponds modification of the denaturation temperature which is significantly different when the protein is incubated with only DMSO. “NI” means that no inhibition was detected. Ki have been determined with GraphPad Prism software using the mixed model inhibition. For asymmetric molecules, the less substituted phenyl ring (*i.e.* the left one on the drawing) is named **B** and the more substituted one (right) is named **A**.

**
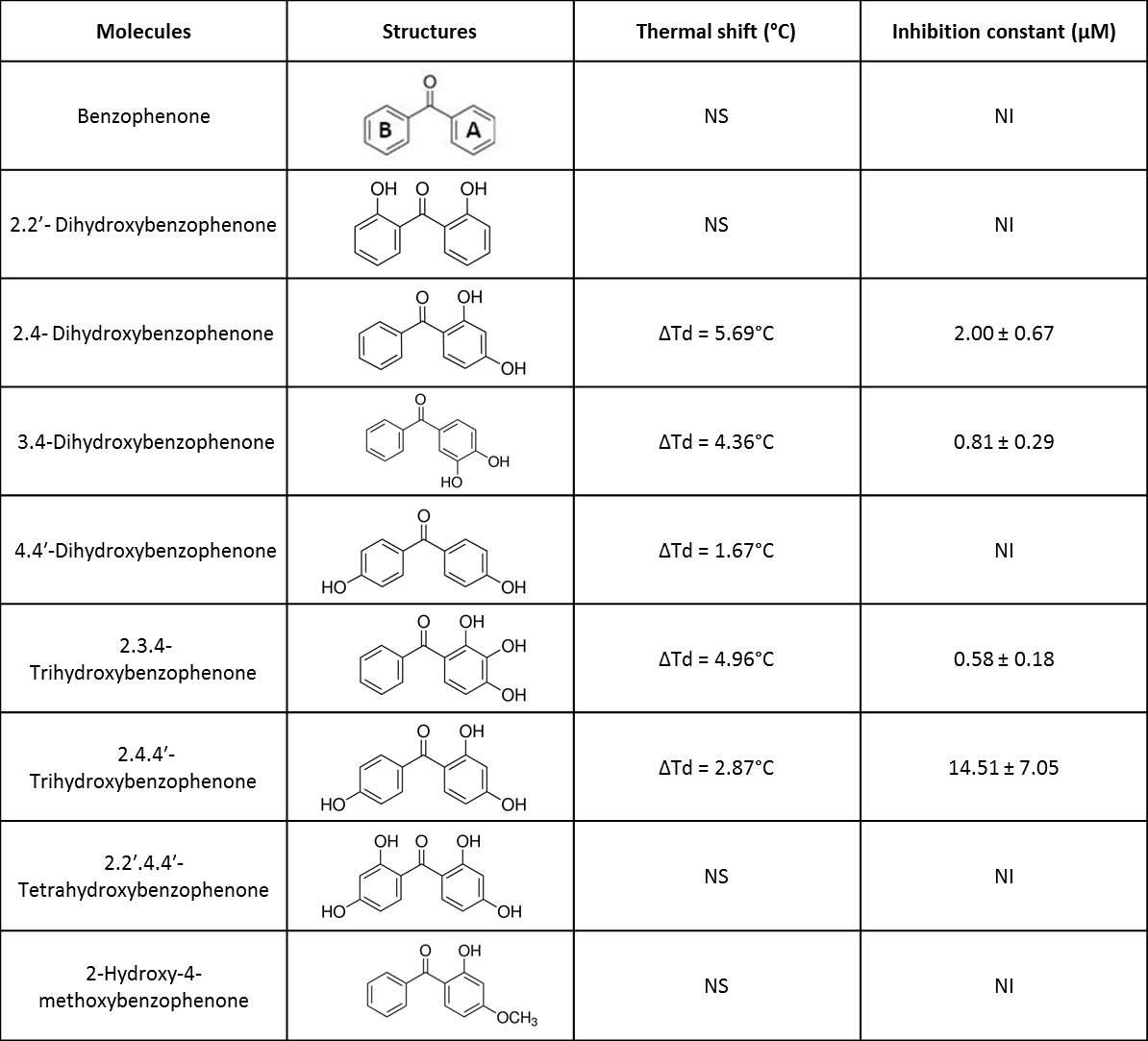
**

Table S4. Effects of several flavonoids on the thermostability of TvGSTOSs

Thermostability was analysed by using 10 µM of protein. The final concentration of the tested molecules was 100 µM in each well. A ∆Td value is only given if the denaturation temperature is significantly modified in the presence of compounds, with respect to incubation with DMSO only. “NS” means that the denaturation temperature has not changed significantly.

**
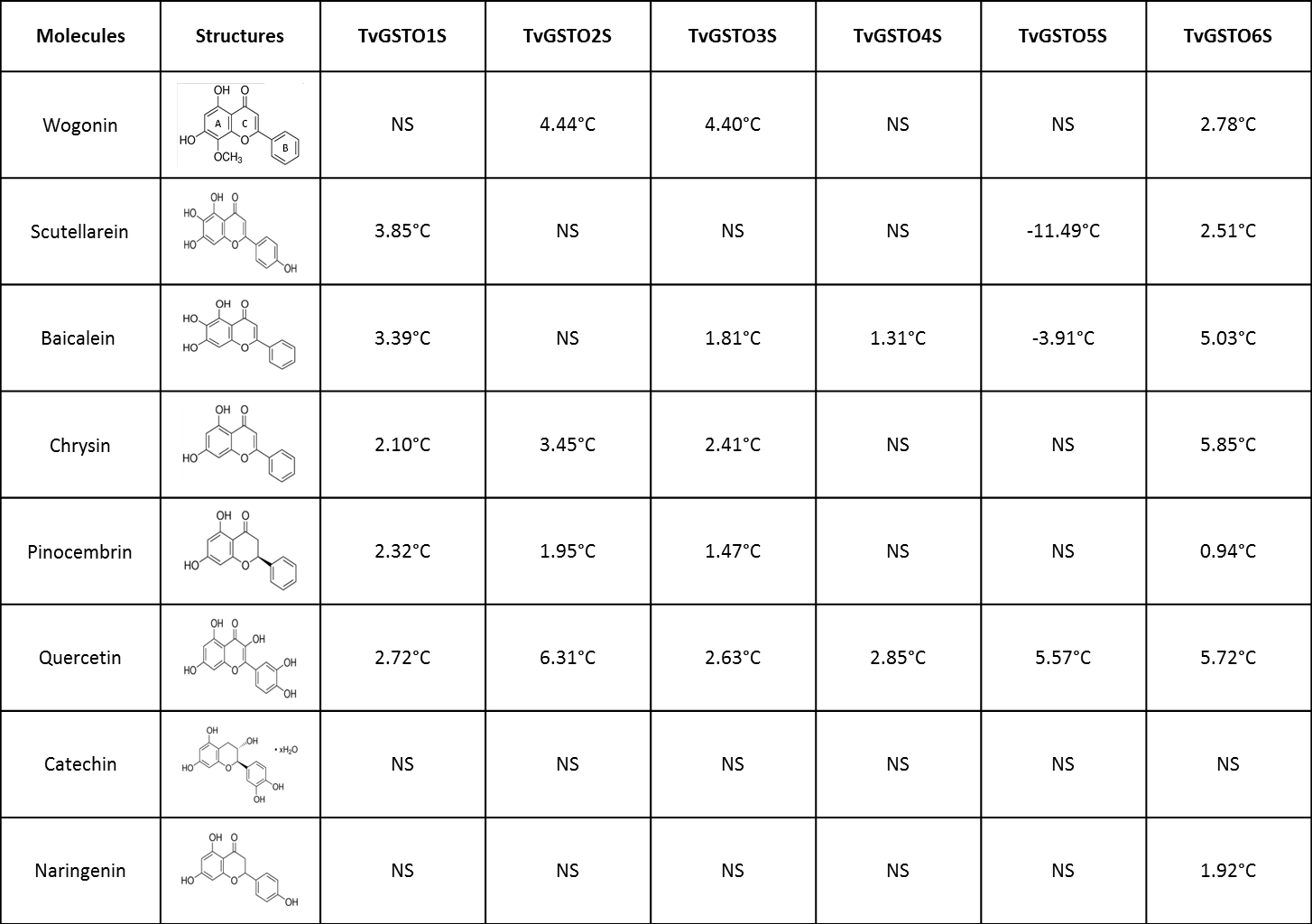
**

Table S5. Inhibition constants (Ki) of TvGSTO3S and TvGSTO6S by wogonin and naringenin

Ki have been determined with GraphPad Prism software using the mixed model inhibition. “NI” means that no inhibition was detected.


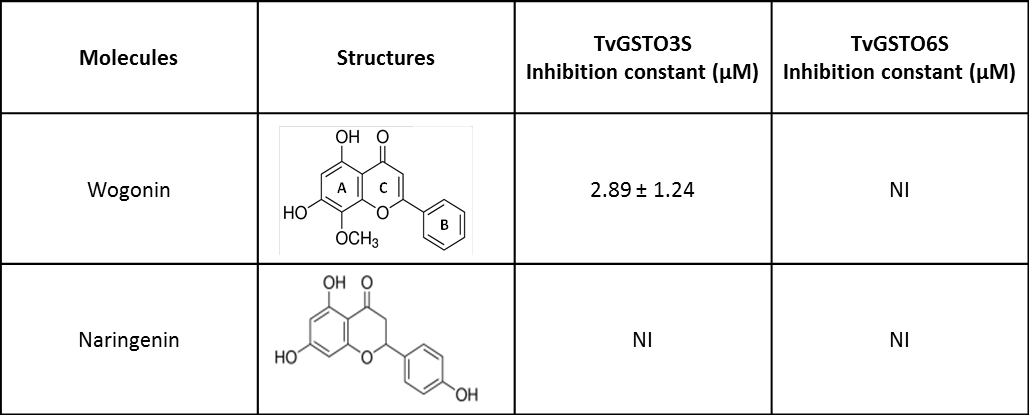


Table S6. Stereo images of 2mFo-DFc electron density maps for each of the eleven structures solved in this study

For each panel, stereo images of a portion of 2mFo-DFc electron density maps are shown to assess quality of the structural data (level at 1.0 σ). Residues from TvGSTO3S structures (F14, S15, P16, Y17, F128 and Y175) and TvGSTO6S structures (A14, S15, P16, F17, F219 and F232) are shown as green sticks.

| 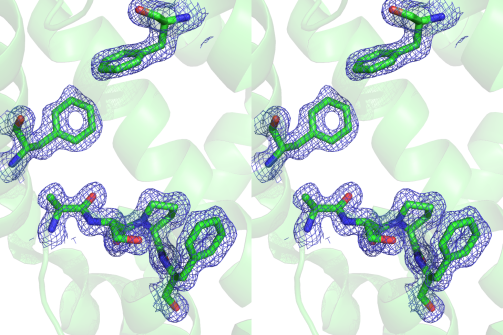  TvGSTO6S apo (1.48 Å) | 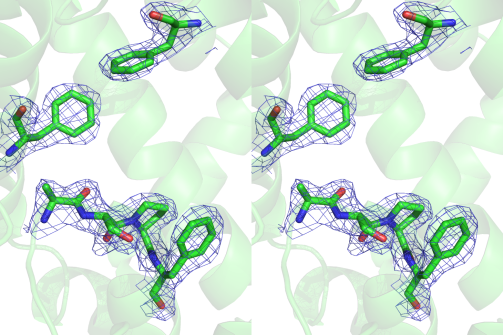  TvGSTO6S - naringenin (2.30 Å) |
| --- | --- |
| 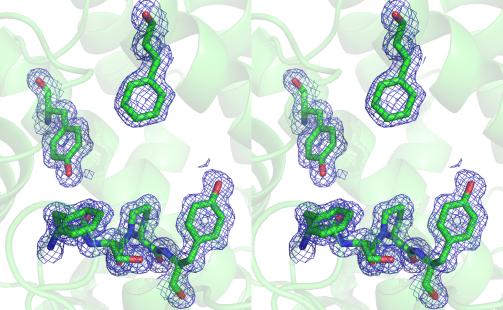  TvGSTO3S apo (1.35 Å) | 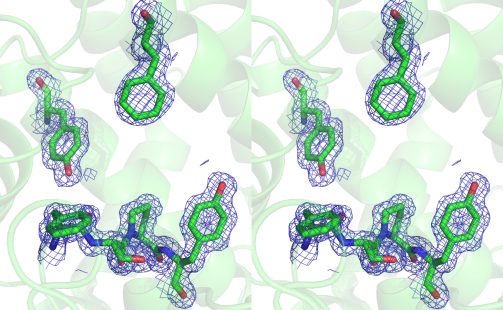  TvGSTO3S - GSH (1.55 Å) |
| 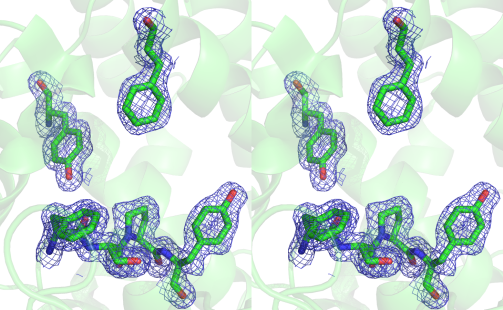  TvGSTO3S - GS-DNB (1.75 Å) | 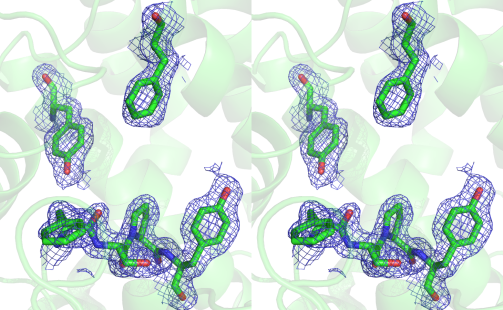  TvGSTO3S - GS-PAP (1.92 Å) |
| 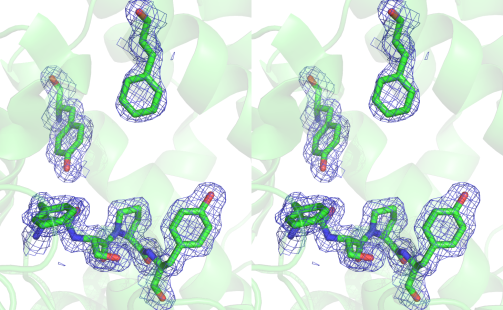  TvGSTO3S - 2,4,4’-HBP (1.70 Å) | 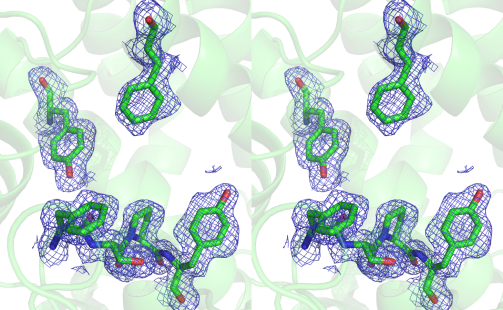  TvGSTO3S - 2,3,4-HBP (1.80 Å) |
| 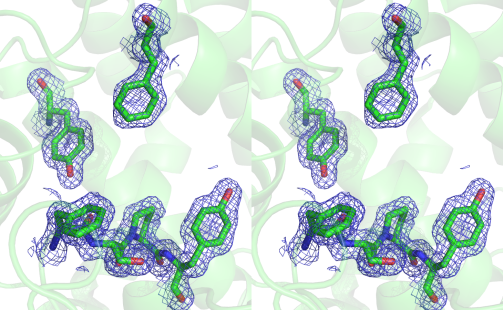  TvGSTO3S - 2,4-HBP (1.75 Å) | 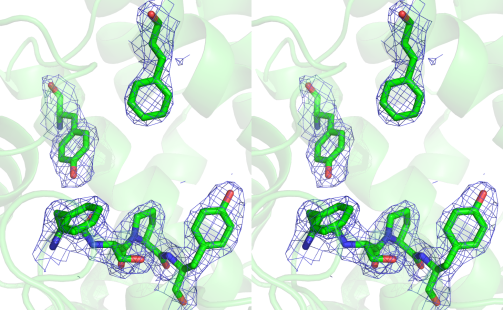  TvGSTO3S - 3,4-HBP (2.40 Å) |
| 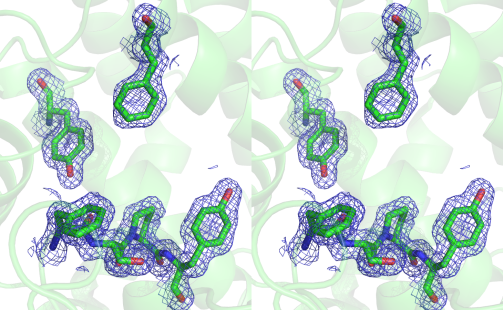  TvGSTO3S - eluate 46-47 (1.70 Å) |  |

References

1 Pei, J. M. & Grishin, N. V. in *Multiple Sequence Alignment Methods* Vol. 1079 *Methods in Molecular Biology* (ed D. J. Russell) 263-271 (2014).

2 Karplus, P. A. & Diederichs, K. Linking crystallographic model and data quality. *Science* **336**, 1030-1033, doi:10.1126/science.1218231 (2012).

3 Tickle, I. J. Experimental determination of optimal root-mean-square deviations of macromolecular bond lengths and angles from their restrained ideal values. *Acta crystallographica. Section D, Biological crystallography* **63**, 1274-1281; author reply 1282-1273, doi:10.1107/S0907444907050196 (2007).

4 Davis, I. W., Murray, L. W., Richardson, J. S. & Richardson, D. C. MOLPROBITY: structure validation and all-atom contact analysis for nucleic acids and their complexes. *Nucleic Acids Res* **32**, W615-619, doi:10.1093/nar/gkh398 (2004).
